# Public Opinions of US Military Medical Research

**DOI:** 10.1001/jamanetworkopen.2026.3875

**Published:** 2026-03-30

**Authors:** Ian H. Stanley, Ian F. Eisenhauer, Steven G. Schauer, Rachel L. Johnson, Franca R. Jones, Christopher J. Button, Michael D. April, Joseph T. Costello, Anne M. Libby, Anne C. Ritter, Vikhyat S. Bebarta

**Affiliations:** 1 Department of Emergency Medicine, University of Colorado Anschutz, Aurora; 2 Center for Combat Medicine and Battlefield (COMBAT) Research, University of Colorado Anschutz, Aurora; 3 Department of Emergency Medicine, Naval Medical Center Portsmouth, Portsmouth, Virginia; 4 Bravo Company, 2nd Medical Battalion, 2nd Marine Logistics Group, Portsmouth, Virginia; 5 US Army Institute of Surgical Research, San Antonio, Texas; 6 Department of Biostatistics and Informatics, University of Colorado Anschutz, Aurora; 7 US Navy Bureau of Medicine and Surgery (BUMED), Falls Church, Virginia; 8 US Air Force Integrated Resilience, Headquarters Air Force, Washington, DC; 9 Department of Military and Emergency Medicine, Uniformed Services University, Bethesda, Maryland; 10 Department of Emergency Medicine, Brooke Army Medical Center (BAMC), San Antonio, Texas; 11 US Army’s 4th Infantry Division, Fort Carson, Colorado

## Abstract

This survey study assesses public awareness of military medical research domains, perceptions of civilian benefit of military medical research, and regional variations.

## Introduction

The US military supports a large medical research enterprise spanning trauma and critical care, prehospital care, psychological health, cancer, infectious diseases, neurodegenerative disorders, and rehabilitation. Beyond sustaining the health and readiness of servicemembers, this portfolio has historically advanced civilian health care.^1^ Examples include establishing tourniquet use to reduce hemorrhage-related morbidity and mortality,^2^ accelerating antibiotic development for infectious diseases,^3^ and pioneering psychological health and suicide prevention strategies.^4^

Public awareness of the scope of and civilian benefit associated with military medical research remains unknown. Understanding public awareness and perceptions may inform translation of dual-use discoveries, clarify funding priorities, and influence the development of military-civilian research partnerships. In this cross-sectional survey study, we assessed awareness of military medical research domains, perceptions of civilian benefit, and regional variations.

## Methods

Participants in this survey study were US adults (≥18 years) recruited through Prolific, an opt-in online survey research platform, using a quota-filling set by Prolific and based on 2021 US Census data distribution of age, sex (female or male), and race (Asian, Black, White, mixed race, or other). Participants viewed a study overview, and informed consent was indicated by their proceeding with a survey. Participants completed a 5-minute online survey (eAppendix in Supplement 1) in October 2025 and were compensated $1.50. We used descriptive statistics and χ^2^ tests, with a 2-sided *P* < .05 considered significant, using R, version 4.5.1. The Colorado Multiple Institutional Review Board approved the study procedures. We followed the AAPOR reporting guideline for survey studies.

## Results

Of 2055 participants (mean [SD] age, 46.2 [15.4] years), 1044 (51.3%) were female and 991 (48.7%) were male (Table 1). Participants were dispersed across the US south (851 [41.6%]), west (471 [23.0%]), midwest (378 [18.5%]), and northeast (345 [16.9%]). Overall, 796 participants (38.8%) reported a familial military service connection, and 100 (4.9%) reported prior personal military service.

**Table 1.** Participants’ Demographic Characteristics (N = 2055)

Characteristic^a^	No./total No. (%)
Age category, y	
18-24	205/2055 (10.0)
25-44	754/2055 (36.7)
45-64	832/2055 (40.5)
≥65	264/2055 (12.8)
Sex	
Female	1044/2035 (51.3)
Male	991/2035 (48.7)
Race and ethnicity^b^^,^^c^	
American Indian or Alaska Native	53/2022 (2.6)
Asian	167/2022 (8.3)
Black or African American	298/2022 (14.7)
Hispanic or Latino	232/2022 (11.5)
Middle Eastern or North African	19/2022 (0.9)
Native Hawaiian or Pacific Islander	14/2022 (0.7)
White	1471/2022 (72.7)
Region of the US	
Northeast	345/2045 (16.9)
Midwest	378/2045 (18.5)
South	851/2045 (41.6)
West	471/2045 (23.0)
Military connection^b^	
Active duty (current)	7/2053 (0.3)
Reserve or National Guard (current)	2/2053 (0.1)
Former military service (retiree or veteran)	100/2053 (4.9)
Family member of service member or retiree or veteran	796/2053 (38.8)
Close friend of service member or retiree or veteran	359/2053 (17.5)
DOD civilian employee or partner	22/2053 (1.1)
No personal military connection	1009/2053 (49.1)
Other	15/2053 (0.7)
Health care professional	107/2049 (5.2)

Abbreviation: DOD, Department of Defense.

^a^ The mean (SD) age of participants was 46.2 (15.4) years. All demographic characteristics were assessed via self-report to characterize the sample.

^b^ Response options not mutually exclusive.

^c^ Race and ethnicity were determined based on respondents’ self-report, using categories published by the US Office of Management and Budget in Statistical Policy Directive No. 15 (SPD 15). Prolific, the recruitment platform, based its US Census quotas on the following categories: Asian, Black, White, mixed race, and other. These differ from the categories shown in this table because our survey used a more nuanced assessment of demographic characteristics.

Awareness that the US military conducts or funds medical research was highest for domains of harmful exposures (1473 [72.0%]), harsh environments (1415 [69.1%]), head injuries (1301 [63.6%]), and burns and severe wounds (1299 [63.6%]) and lowest for suicide prevention (969 [47.2%]) and cancer (550 [26.9%]); across domains, 483 (23.6%) to 997 (48.7%) indicated “I don’t know” (Table 2). Most respondents agreed that military medical research benefits national public health (1522 [74.4%]), generates innovations applicable to civilian health care (1487 [72.7%]), and should be translated for civilian use (1831 [89.3%]). Neither awareness nor perceptions significantly differed by US region. Overall, 1163 respondents (56.6%) indicated a desire to learn more about the civilian benefit associated with military medical research.

**Table 2.** Participants’ Awareness and Perceptions of the Association of US Military Medical Research With Civilian Health Care

Awareness	Total No.	No. (%)		
		Yes	No	I don’t know
Harmful exposures (eg, study effects of chemicals, toxins, radiation, or pollutants)	2045	1473 (72.0)	89 (4.4)	483 (23.6)
Operating in harsh environmental conditions (eg, understand how the body handles heat, cold, or high mountains)	2047	1415 (69.1)	114 (5.6)	518 (25.3)
Burns and severe wounds (eg, repair burns or regrow tissue)	2044	1299 (63.6)	94 (4.6)	651 (31.8)
Head injuries (eg, treat concussions or other brain injuries)	2046	1301 (63.6)	132 (6.5)	613 (30.0)
Rehabilitation (eg, help people regain movement or function after injury)	2045	1259 (61.6)	184 (9.0)	602 (29.4)
Mental health (eg, treat PTSD or depression)	2040	1215 (59.6)	251 (12.3)	574 (28.1)
Remote care (eg, deliver medical treatment in environments without hospitals, such as battlefields)	2050	1167 (56.9)	179 (8.7)	704 (34.3)
Blood loss and shock (eg, stop heavy bleeding and help the body recover after severe injury)	2045	1153 (56.4)	110 (5.4)	782 (38.2)
Infections (eg, stop malaria or other dangerous germs)	2040	1128 (55.3)	202 (9.9)	710 (34.8)
Suicide prevention (eg, help individuals cope with suicidal thoughts)	2051	969 (47.2)	293 (14.3)	789 (38.5)
Cancer (eg, find new treatments to stop cancer from growing or spreading)	2047	550 (26.9)	500 (24.4)	997 (48.7)
**Perceptions**		**Agree**	**Neutral**	**Disagree**
Military medical research delivers valuable advancements to the nation’s public health infrastructure.	2047	1522 (74.4)	393 (19.2)	132 (6.4)
Innovations from military medical research translate into health benefits for the general public.	2044	1487 (72.7)	368 (18.0)	189 (9.2)
The benefits of military medical research are only for service members and veterans.	2048	460 (22.5)	322 (15.7)	1266 (61.8)
Military medical research has accelerated advancements in emergency medical care for the general public.	2046	1391 (68.0)	462 (22.6)	193 (9.4)
Military medical research has accelerated advancements in infectious diseases for the general public.	2045	1039 (50.8)	743 (36.3)	263 (12.9)
Military medical research has accelerated advancements in mental health for the general public.	2051	903 (44.0)	707 (34.5)	441 (21.5)
Collaborations between military and academic institutions strengthen the quality of medical research.	2053	1639 (79.8)	337 (16.4)	77 (3.8)
The US military and civilian partners should collaborate more closely on medical research.	2048	1726 (84.3)	260 (12.7)	62 (3.0)
Findings from military medical research should be translated for use in the general population.	2051	1831 (89.3)	184 (9.0)	36 (1.8)

Abbreviation: PTSD, posttraumatic stress disorder.

## Discussion

In this national survey, many adults were aware of and expressed favorable views toward US military medical research. Awareness was highest for domains with operational relevance (ie, harmful exposures, harsh environments, head injuries, and burns), suggesting public association of military medical research with combat-related or visible injuries, rather than chronic or less visible health conditions, such as cancer or suicide prevention. Nevertheless, the high proportion of “I don’t know” responses across the 11 medical research domains assessed, nearly 50% for some domains, indicates limited familiarity with the scope of US military medical research.

Approximately 90% of respondents agreed that military medical research innovations should be translated for civilian use, underscoring appreciation for their broad applicability. Awareness and perceptions were consistent across US regions, suggesting positive public sentiment toward continued military-civilian collaboration and transition of military-developed health solutions into civilian health care. Strengthening military-civilian research partnerships and translations will likely also extend benefits to other important areas, such as combat medical training^5^ and medical disaster response preparedness.^6^

The high prevalence of uncertain or inaccurate awareness—together with strong public support for and interest in learning more about military medical research—highlights opportunities for improved public-facing communication. Study limitations include lack of determinable response rate and use of nonprobability sampling of individuals who opted into a research survey platform, potentially yielding overestimates.

## Notes

Supplement 1. **eAppendix.** Measure Used to Assess Public Awareness and Perceptions of US Military Medical Research

Supplement 2.
Data Sharing Statement

## References

[1] Kellermann AL, Kotwal RS, Rasmussen TE. Military medicine’s value to US health care and public health: bringing battlefield lessons home. JAMA Netw Open. 2023;6(9):e2335125. doi:10.1001/jamanetworkopen.2023.35125 37733341

[2] Goodwin T, Moore KN, Pasley JD, Troncoso R Jr, Levy MJ, Goolsby C. From the battlefield to main street: tourniquet acceptance, use, and translation from the military to civilian settings. J Trauma Acute Care Surg. 2019;87(1S)(suppl 1):S35-S39. doi:10.1097/TA.0000000000002198 31246904

[3] Hospenthal DR. History of U.S. military contributions to the understanding, prevention, and treatment of infectious diseases: an overview. Mil Med. 2005;170(4)(suppl):1-2. doi:10.7205/MILMED.170.4S.1 15916277

[4] Stanley IH, Embrey EP, Bebarta VS. Actualizing military suicide prevention through digital health modernization. JAMA Psychiatry. 2024;81(12):1173-1174. doi:10.1001/jamapsychiatry.2024.2679 39320870

[5] Grabo DJ, Gurney JM, Parascandola L, Knudson MM. Military-civilian partnerships and the clinical readiness mission: a preliminary study from the Military Health System and the American College of Surgeons. J Trauma Acute Care Surg. 2022;93(2S suppl 1):S16-S21. doi:10.1097/TA.0000000000003704 35583979

[6] Goralnick E, Holcomb JB, Elster EA. Preparing the civilian health care system for wartime: a national imperative for military-civilian integration. JAMA. 2025;334(19):1703-1704. doi:10.1001/jama.2025.18642 41129137

