# Supplementary material for: Public Opinions of US Military Medical Research
Source: JAMA Netw Open. 2026 Mar 30;9(3):e263875. doi: 10.1001/jamanetworkopen.2026.3875 (PMC13036569; doi:10.1001/jamanetworkopen.2026.3875)
Supplement: Supplement 1. — eAppendix. Measure Used to Assess Public Awareness and Perceptions of US Military Medical Research [file jamanetwopen-e263875-s001.pdf]

Supplementary Online Content

Stanley IH, Eisenhauer IF, Schauer SG, et al. Public opinions of US military medical research. *JAMA Netw Open.* 2026;9(3):e263875. doi:10.1001/jamanetworkopen.2026.3875

eAppendix. Measure Used to Assess Public Awareness and Perceptions of US Military Medical Research

This supplementary material has been provided by the authors to give readers additional information about their work.

© 2026 Stanley IH et al. *JAMA Network Open.*

eAppendix. Measure Used to Assess Public Awareness and Perceptions of US Military Medical Research

The following questions and response options were presented to participants to assess the domains presented in the accompanying manuscript.

To the best of your knowledge, does the U.S. Department of Defense fund or conduct medical research in the following areas:

	Yes	No	I don't know
**Blood Loss & Shock** (e.g., stop heavy bleeding and help the body recover after severe injury)			
**Burns & Severe Wounds** (e.g., repair burns or regrow tissue)			
**Cancer** (e.g., find new treatments to stop cancer from growing or spreading)			
**Harmful Exposures** (e.g., study effects of chemicals, toxins, radiation, or pollutants)			
**Head Injuries** (e.g., treat concussions or other brain injuries)			
**Infections** (e.g., stop malaria or other dangerous germs)			
**Mental Health** (e.g., treat PTSD or depression)			
**Operating in Harsh Environmental Conditions** (e.g., understand how the body handles heat, cold, or high mountains)			
**Rehabilitation** (e.g., help people regain movement or function after injury)			
**Remote Care** (e.g., deliver medical treatment in environments without hospitals, such as battlefields)			
**Suicide Prevention** (e.g., help individuals cope with suicidal thoughts)			

© 2026 Stanley IH et al. *JAMA Network Open.*

We are interested in learning more about the public's views on U.S. military medical research. For the purposes of this survey, we offer the following definitions:

- **Military medical research:** scientific and clinical research that is conducted or funded by the U.S. Department of Defense (DoD). This is separate from research conducted by the U.S. Department of Veterans Affairs (VA).
- **General public:** individuals not currently serving in the U.S. military.

Please indicate how much you agree or disagree with each of the following statements using the provided scale.

	Strongly disagree	Disagree	Neither agree nor disagree	Agree	Strongly agree
Military medical research delivers valuable advancements to the nation's public health infrastructure.					
Innovations from military medical research translate into health benefits for the general public.					
The benefits of military medical research are only for service members and veterans.					
Military medical research has accelerated advancements in **emergency medical care** for the general public.					
Military medical research has accelerated advancements in **infectious diseases** for the general public.					
Military medical research has accelerated advancements in **mental health** for the general public.					
Choose the first option-"strongly disagree"-in answering this question.					
Collaborations between military and academic institutions strengthen the quality of medical research.					
The U.S. military and civilian partners should collaborate more closely on medical research.					
Findings from military medical research should be translated for use in the general population.					

[*Methodology Note: For analyses, we collapsed response options into three categories of disagree, neutral, or agree.*]

Would you like to learn more about military medical research and its benefits to the general public?

- ☐ Yes
- ☐ No

© 2026 Stanley IH et al. *JAMA Network Open.*
