# Supplementary material for: Public Opinions of US Military Medical Research
Source: JAMA Netw Open. 2026 Mar 30;9(3):e263875. doi: 10.1001/jamanetworkopen.2026.3875 (PMC13036569; doi:10.1001/jamanetworkopen.2026.3875)
Supplement: Supplement 2. — Data Sharing Statement [file jamanetwopen-e263875-s002.pdf]

Data Sharing Statement

Stanley. Public Opinions of US Military Medical Research. *JAMA Netw Open*. Published March 30, 2026. doi:10.1001/jamanetworkopen.2026.3875

Data
Data available: No
